# Dissection of transcriptome dysregulation and immune characterization in women with germline BRCA1 mutation at single-cell resolution

**DOI:** 10.1186/s12916-022-02489-9

**Published:** 2022-09-09

**Authors:** Xuexin Yu, Wanrun Lin, Alexandra Spirtos, Yan Wang, Hao Chen, Jianfeng Ye, Jessica Parker, Ci Ci Liu, Yiying Wang, Gabriella Quinn, Feng Zhou, Setsuko K. Chambers, Cheryl Lewis, Jayanthi Lea, Bo Li, Wenxin Zheng

**Affiliations:** 1 Lyda Hill Department of Bioinformatics, UT Southwestern Medical Center, Dallas, TX USA; 2 Department of Pathology, University of Texas Southwestern Medical Center, Dallas, TX USA; 3 Department of Obstetrics and Gynecology, University of Texas Southwestern Medical Center, Dallas, TX USA; 4 Present address: Department of Obstetrics and Gynecology, Indiana University, Indianapolis, IN USA; 5 Present address: Department of Obstetrics and Gynecology, University of Rochester Medical Center, Rochester, NY USA; 6 Present address: Department of Pathology, Women’s Hospital, School of Medicine, Zhejiang University, Hangzhou, China; 7 Department of Obstetrics and Gynecology, The University of Arizona Cancer Center, University of Arizona, Tucson, AZ USA; 8 Harold C. Simmons Comprehensive Cancer Center, UT Southwestern Medical Center, Dallas, TX USA; 9 Department of Immunology, UT Southwestern Medical Center, Dallas, TX USA

**Keywords:** High-grade serous carcinoma, Ovarian cancer, BRCA1 mutation, Fallopian tube, Single-cell RNA sequencing, T cell exhaustion, Epithelial to mesenchymal transition

## Abstract

**Background:**

High-grade serous carcinoma (HGSC) is the most frequent and lethal type of ovarian cancer. It has been proposed that tubal secretory cells are the origin of ovarian HGSC in women with familial *BRCA1*/*2* mutations. However, the molecular changes underlying malignant transformation remain unknown.

**Method:**

We performed single-cell RNA and T cell receptor sequencing of tubal fimbriated ends from 3 *BRCA1* germline mutation carriers (*BRCA1* carriers) and 3 normal controls with no high-risk history (non-*BRCA1* carriers).

**Results:**

Exploring the transcriptomes of 19,008 cells, predominantly from *BRCA1*^+^ samples, we identified 5 major cell populations in the fallopian tubal mucosae. The secretory cells of *BRCA1*^+^ samples had differentially expressed genes involved in tumor growth and regulation, chemokine signaling, and antigen presentation compared to the wild-type BRCA1 controls. There are several novel findings in this study. First, a subset of the fallopian tubal secretory cells from one *BRCA1* carrier exhibited an epithelial-to-mesenchymal transition (EMT) phenotype, which was also present in the mucosal fibroblasts. Second, we identified a previously unreported phenotypic split of the EMT secretory cells with distinct evolutionary endpoints. Third, we observed increased clonal expansion among the CD8^+^ T cell population from *BRCA1*^+^ carriers. Among those clonally expanded CD8^+^ T cells, PD-1 was significantly increased in tubal mucosae of *BRCA1*^+^ patients compared with that of normal controls, indicating that T cell exhaustion may occur before the development of any premalignant or malignant lesions.

**Conclusion:**

These results indicate that EMT and immune evasion in normal-looking tubal mucosae may represent early events leading to the development of HGSC in women with *BRCA1* germline mutation. Our findings provide a probable molecular mechanism explaining why some, but not all, women with *BRCA1* germline mutation present with early development and rapid dissemination of HGSC.

**Supplementary Information:**

The online version contains supplementary material available at 10.1186/s12916-022-02489-9.

## Background

Ovarian epithelial cancer (OEC) is the most lethal gynecologic malignancy in the USA. The most common histological subtype of OEC is high-grade serous carcinoma (HGSC); the vast majority of these (>80%) are believed to arise in the fallopian tube [1]. HGSC are predominantly (80%) diagnosed at advanced stages (III–IV), with a 5-year survival rate of approximately 30%, resulting in the largest number of ovarian cancer-associated deaths [2]. Diagnosis of early-stage disease improves the 5-year survival of patients with HGSC to over 90%. Unfortunately, despite significant efforts to identify reliable tools for ovarian cancer screening in the last several decades, there are no effective early detection methods for HGSC [3].

Hereditary ovarian cancer can occur through mutations in many different genes; up to 23% of OECs have been found to occur in patients with germ-line mutations [4]. Patients with a *BRCA* mutation are particularly susceptible to developing OEC. *BRCA1/2* mutations occur in 1 in 300–500 women and together account for 15–17% of ovarian high-grade serous carcinoma (HGSC) cases [5, 6]. Compared with *BRCA2*, inherited *BRCA1* mutations confer a higher lifetime risk of developing OEC (~39% vs. ~11%) [7–10]. Studies performed on ovaries and fallopian tubes after prophylactic salpingo-oophorectomy in patients with *BRCA* mutations revealed the presence of clinically undetected, occult cancers in the fallopian tubes, with histologically normal ovaries without precancerous lesions [11–13]. Subsequent studies showed that precancerous and/or latent precancerous lesions including serous tubal intraepithelial carcinoma (STIC), serous tubal intraepithelial lesions (STIL), secretory cell outgrowth (SCOUT), secretory cell expansion (SCE), and p53 signatures are frequently detected in the distal portion of the fallopian tube epithelia (FTE), but not in the ovary [14–18]. It is believed that these (latent) precancerous lesions may arise from secretory cells, one of the two major cell types within the FTE [19, 20]. Collectively, these findings suggest that the FTE is likely the cellular origin of the ovarian HGSC.

In order to elucidate the genetic changes of HGSC development, researchers have used high-throughput bulk sequencing to identify essential genes in the process. Using CHIP-seq and transcriptomic studies, Lawrensen et al. found that the transcription factor SOX18, which was overexpressed in tumors, induced an EMT in FTEs from patients with HGSC [21], suggesting that an EMT in FTE may represent an early molecular mechanism of HGSC development. In addition to EMT, many other genetic changes are found within the FTE of BRCA mutation carriers [22, 23]. Many previous studies addressing BRCA germline mutation-associated etiologies are bulk sequencing or tissue based, and therefore unable to identify subpopulations within the FTE since all cells derived from FTE were evaluated together as a single sample. The development of single-cell RNA sequencing (scRNA-seq) has enabled the distinction of individual cellular subtypes in complex tissues. Using this approach, researchers have identified distinct cellular subtypes in FTE and correlated them to a different level of OEC differentiation. Hu et al. described six subtypes of FTE cells in normal human fallopian tube tissue. This included an “EMT subtype” that represented a presumed partial epithelial-mesenchymal transition. They then used these subtypes to profile HGSC samples and identify associations with patient prognosis. Tumors enriched in cells from the EMT subtype (“EMT-high”) were associated with poor overall survival [24]. These “Tumor initiating cells” with mesenchymal and stem cell features have also been identified in ovarian cancer. Such tumor initiation process is linked to EMT in *BRCA1*^*+*^ breast cancer, but this has not yet been investigated in ovarian cancer [25]. Furthermore, cancer cells undergoing EMT, or transitioning along the EMT spectrum, may escape immune surveillance through multiple mechanisms, whereby shaping the tumor microenvironments and decreasing susceptibility to immune effector cells [26]. It is known that tumor cells of HGSC have various molecular mechanisms to bypass the body’s immune monitoring function. This typically happens in advanced stages [27]. In addition, there are no studies addressing the immune composition of tubal tissue from individuals with *BRCA1* germline mutations. Alterations in the immune composition of these tissues could provide insight into the role of immune surveillance involved in the development of HGSC. Identifying such changes prior to the development of precancerous or cancerous lesions could also lead to the development of more effective screening and immunotherapy targets.

The essential genetic changes that occur in FTE in women with germline *BRCA1/2* mutations remain elusive at the single-cell level. Here, we investigated the differences between the FTE of BRCA1 mutation carriers and normal controls at the cellular and molecular level by identifying the essential transcriptional changes of the tubal mucosal cells using deep single-cell RNA-seq techniques. This study addressed the following questions to explore the molecular changes related to the initiation of HGSC. (1) What are the major differences in gene expression within the FTE between *BRCA1* carriers and non-carriers? (2) Are there any cancer-prone activities within the mesenchymal cells of the tubal mucosae in *BRCA1* carriers? (3) Is there any immune-escape-related mechanism involving normal-looking tubal mucosae in patients with *BRCA1* carriers?

## Methods

### Institutional review board approval and collection of human tissue samples

Patients undergoing risk-reducing salpingo-oophorectomy for a positive *BRCA1* germline mutation between March 2019 and November 2019 were identified. The *BRCA1* mutations were confirmed using next-generation sequencing analysis performed by commercially available genetic testing after the patient had undergone genetic counseling. Age-matched controls were selected from patients between 35 and 49 years old who had salpingo-oophorectomy performed for benign indications (leiomyoma, abnormal uterine bleeding). Informed consent was obtained from all patients before surgery. Tubal epithelia were collected intraoperatively using a standard procedure previously described [28, 29]. Briefly, a cytobrush was introduced into the fallopian tube lumen and rotated gently three times before being withdrawn across the fimbriated surface. The cytobrush containing obtained tubal epithelial cells was agitated in Dulbecco’s modified Eagle’s medium (Thermo Fischer, catalog #-11966025). The samples were immediately processed for RNA-seq. In addition, a representative sample of the distal end of the fallopian tubal was collected from 3 patients with BRCA1 germline mutation and 3 age-matched patients. All samples were confirmed to have a histological diagnosis of normal ovaries and fallopian tubes without evidence of carcinoma or serous tubal intraepithelial carcinoma.

### Single-cell library preparation

Single-cell RNA-seq was performed by the 10× Genomic single-cell 5′ VDJ library platform. The concentration of single-cell suspensions was adjusted to 900-1100 cells/μl. Cells were loaded between 10,000 and 17,000 cells/chip position using the Chromium Single-cell 5′ VDJ Library, Gel Bead & Multiplex Kit, and Chip Kit (10× Genomics, V1 barcoding chemistry). Single-cell gene expression and TCR libraries were generated according to the manufacturer’s instructions. All subsequent steps were performed following the manufacturer’s standard protocols. Purified libraries were analyzed by Novaseq sequencer with 150-bp paired-end reads at a targeted median read depth of 50,000 reads per cell from total gene expression libraries and 5000 reads per cell for TCR libraries.

### Batch effect and quality control of single-cell RNA-seq and scTCR data

**Fig. 1:**
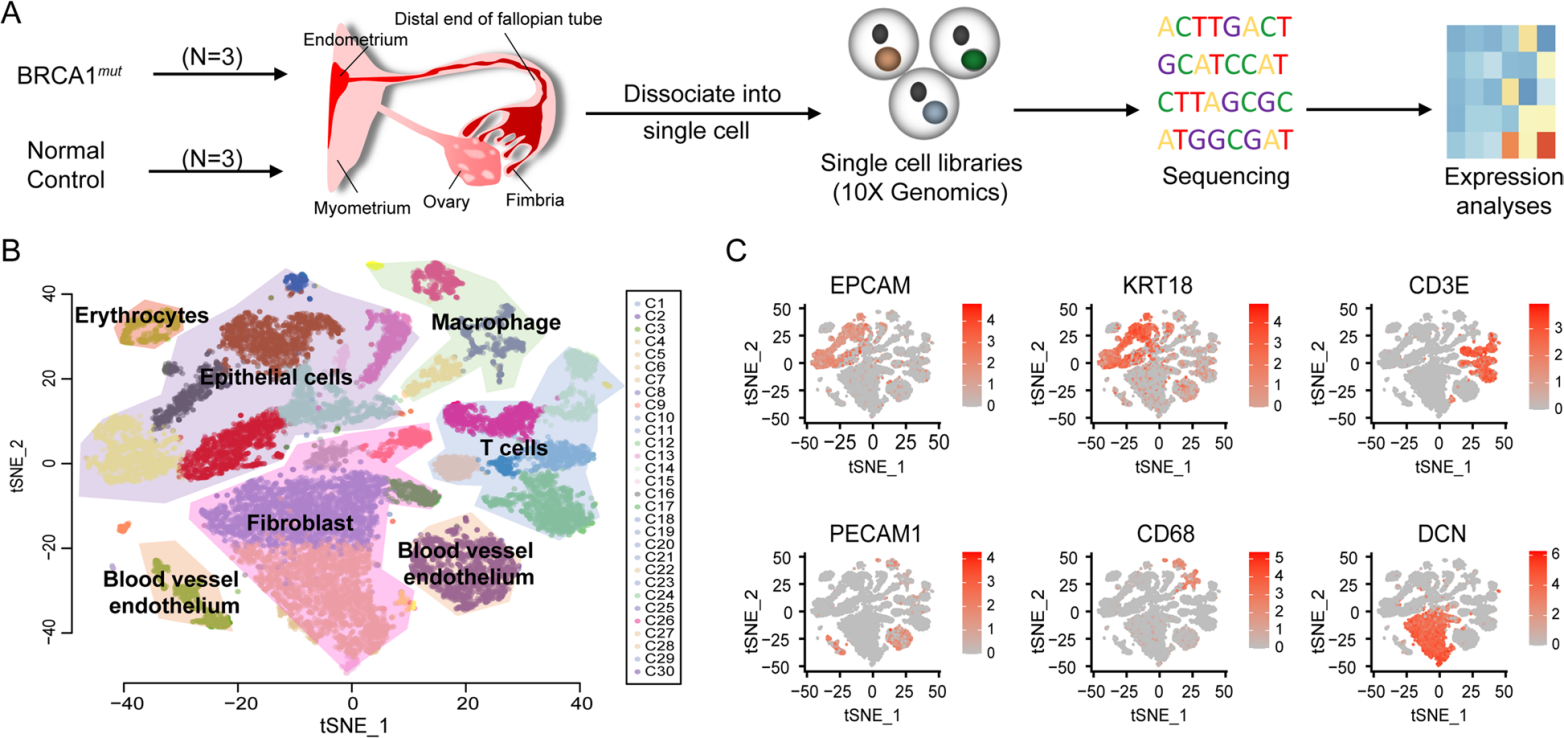
Single-cell transcriptome profiling of the tubal fimbriated end. **A** Scheme of single-cell transcriptome experiment, from tissue sample collection to data visualization. **B** Two-dimensional tSNE projection of 19,008 cells from 6 samples, showing the formation of 5 main cell types, including epithelial cells, fibroblasts, T cells, macrophages, and blood vessel endothelial cells. **C** Expression of the marker genes for 5 major cell types; cell positions are from the tSNE plot in B

The analysis pipeline in Cell Ranger version 3.0.1 was used for raw sequencing data processing. This was used to align to the GRCh38 reference genome, filter, and quantify. The output of *cellranger aggr* was loaded into R by using an R package, Seurat (version 3.0.1). After removing cells with high mitochondrial content (>= 15%), 19,008 cells were kept for downstream analysis. All samples were sequenced in two-time points. We used Seurat for data integration and alignment using canonical correlation analysis (CCA) to remove the batch effect. After batch effect correction, we found the mixture of cells from distinct samples in all clusters of Fig. 1B (Fig. S1). Most of these clusters contained cells from 4 to 6 samples. In addition, there are four rear cell populations were composed of cells from less than four samples, including Cluster 21 (cells came from 3 samples), Cluster 24 (cells came from 2 samples), Cluster 27 (cells came from 2 samples), and Cluster 28(cells came from 3 samples). For scTCR-seq data, TCR reads were aligned to the GRCh38 reference genome and TCR annotation was performed using the 10× cellranger vdj pipeline. The Seurat R package was also used for cell clustering and tSNE visualization.

### Differential gene expression analyses

After normalization, the data matrix contained 33,538 genes. Significantly differentially expressed genes were identified using the eBayes function in limma R package, comparing all the BRCA1 mutation carriers with normal controls as the baseline. We adjusted *p* values (*q* values) for multiple testing using the Benjamini–Hochberg method. The differentially expressed genes (*q* < 0.05) with an absolute log_2_ fold change (FC) > 0.2 were identified as upregulated genes, while the absolute log_2_ fold change (FC) < 0.2 were identified as downregulated genes. All differentially expressed genes were ranked by log_2_ fold change. Pathway enrichment was performed on ranked lists with gene set enrichment analysis (GSEA) using KEGG.

### Characterization of EMT-TFs enrichment

We used the ratio of observed to expected cell number expressed specific EMT-TF in *BRCA1* mutation carriers and normal controls to measure the EMT-TF enrichment. Given a contingency table of one EMT-TF across different samples, we performed a Chi-squared test to evaluate whether the distribution of EMT-TF across different samples significantly deviates from random expectations. The extent of deviation for each combination of EMT-TF and sample is quantified by the ROE value:

$$\mathrm{ROE}=\frac{N_o}{N_e}$$

where *N*_*o*_ is the observed number cell for a specific EMT-TF and sample combination, and *N*_*e*_ is the expected number calculated from the Chi-squared test. If ROE > 1, it means that the cells expressing specific EMT-TF in one sample type are more frequent than would be seen with random expression for the analyzed samples and vice versa.

### Cluster similarity analysis

To distinguish distinct cell populations, a clustering approach was used as previously described by Hu et al [24]. There are 15 epithelial clusters in our data, with the smallest cluster being made up 32 cells. Variation in cluster sizes can affect the similarity score of two clusters, so we randomly selected 20 cells from each epithelial cluster and combined the expression data of these cells together. Based on this expression profile, we used the findMutualNN function in batchelor R package to identify all cell pairs between our epithelial clusters and Hu’s four secretory cells subtypes. We then summarized the number of cell pairs of each epithelial cluster for four secretory cells subtypes and calculated the ratio of the number of pairs between two clusters to the number of pairs overall. The ratio was used as a similarity score.

### Single-cell pseudotime trajectory analysis

We used Monocle2 to determine the single-cell pseduotime trajectory (http://cole-trapnell-lab.github.io/monocle-release/tutorials/). Monocle object was formed by Monocle implemented newCellDataSet function with lowerDetectionLimit=0.5.

### RNA velocity-based cell fate tracing

To perform the RNA velocity analysis, notation of spliced and unspliced reads was performed using Python script velocyto.pyon Cell Ranger output folder. RNA velocity was estimated using the gene-relative model with k-nearest neighbor cell pooling (*k* = 20). For other parameters, we used the standard R implementation of velocyto with default settings.

### Immunohistochemistry (IHC)

Differentially expressed interested biomarkers (immune and EMT related) were validated by using IHC single and dual stains in the fallopian tube sections from patients with or without *BRCA1* mutations. Formalin-fixed, paraffin-embedded tissues were cut to a thickness of 4 μm. Single-marker staining was performed on sections according to a standard immunostaining protocol using a Ventana Benchmark XT Automated Stainer, following the manufacturer’s instructions. Briefly, the sections were heated for 1 hour at 58°C, deparaffinized, and dehydrated in a graded alcohol series. Antigen retrieval was performed using Cell Conditioning 1 (Ventana) Tris/Borate/EDTA for 60 minutes with a Ventana Benchmark XT Automated Stainer followed by either single or dual-marker immunostaining, according to the manufacturer’s instructions. The sections were blocked with hydrogen peroxide and normal goat serum and then incubated for 30 minutes at 37°C. The color was developed with 3,3′-diaminobenzidine (DAB) and/or alkaline phosphatase, and the sections were then counterstained with hematoxalin. Appropriate positive and negative controls were included in every batch of immunostaining. All slides were reviewed independently by 3 pathologists (WL, YaW, and WZ) who were blinded to the clinical data. For all stains, only moderate or strong intensity of the IHC staining was considered as positive, while absent or weak stains were considered as negative.

## Results

### Single-cell transcriptome profiling of the fallopian tube from BRCA1 and non- BRCA1 carriers

Fresh tissue from the tubal fimbriated end from 3 *BRCA1* carriers (BRCA1_1, 1_2, and 1_3) and 3 *BRCA1* wild-type patients (Normal_1, _2, and_3) was profiled by 10× genomics single-cell RNA sequencing technology (Fig. 1A and Table S1). In total, we obtained 19,008 cells, with a mean of 2213 genes detected per cell. 11,999 cells were obtained from the *BRCA1* carriers, while 7029 cells were from non-*BRCA1* carriers. Cells with over 15% mitochondrial gene expression were removed prior to the analysis. All remaining cells were pooled and visualized using t-distributed stochastic neighbor embeding (t-SNE) (Fig. 1B). Applying unsupervised graph-based clustering, we identified 30 clusters covering 5 major cell types within the fallopian tube (Fig. 1B); the same t-SNE plot of cells colored by different samples was represented in Fig. S1. These cell types included epithelial cells (EPCAM^+^ and KRT18^+^); fibroblasts (decorin, or DCN^+^); T cells with CD3 expression; macrophages (CD68^+^); and blood vessel endothelium characterized by platelet and endothelial cell adhesion molecule 1 (PECAM1) expression (Fig. 1C). We summarized the cell number and percentage of each major cell type in each patient in Table S2. The top 100 biomarkers (Table S3) of each major cell type are highly consistent with the literature reported cell identities [24, 30], suggesting that the experimental protocol faithfully captured the mucosal constituents of the fallopian tubal samples.

### Transcriptome dysregulation of secretory and ciliated cells in BRCA1 carriers

**Fig. 2:**
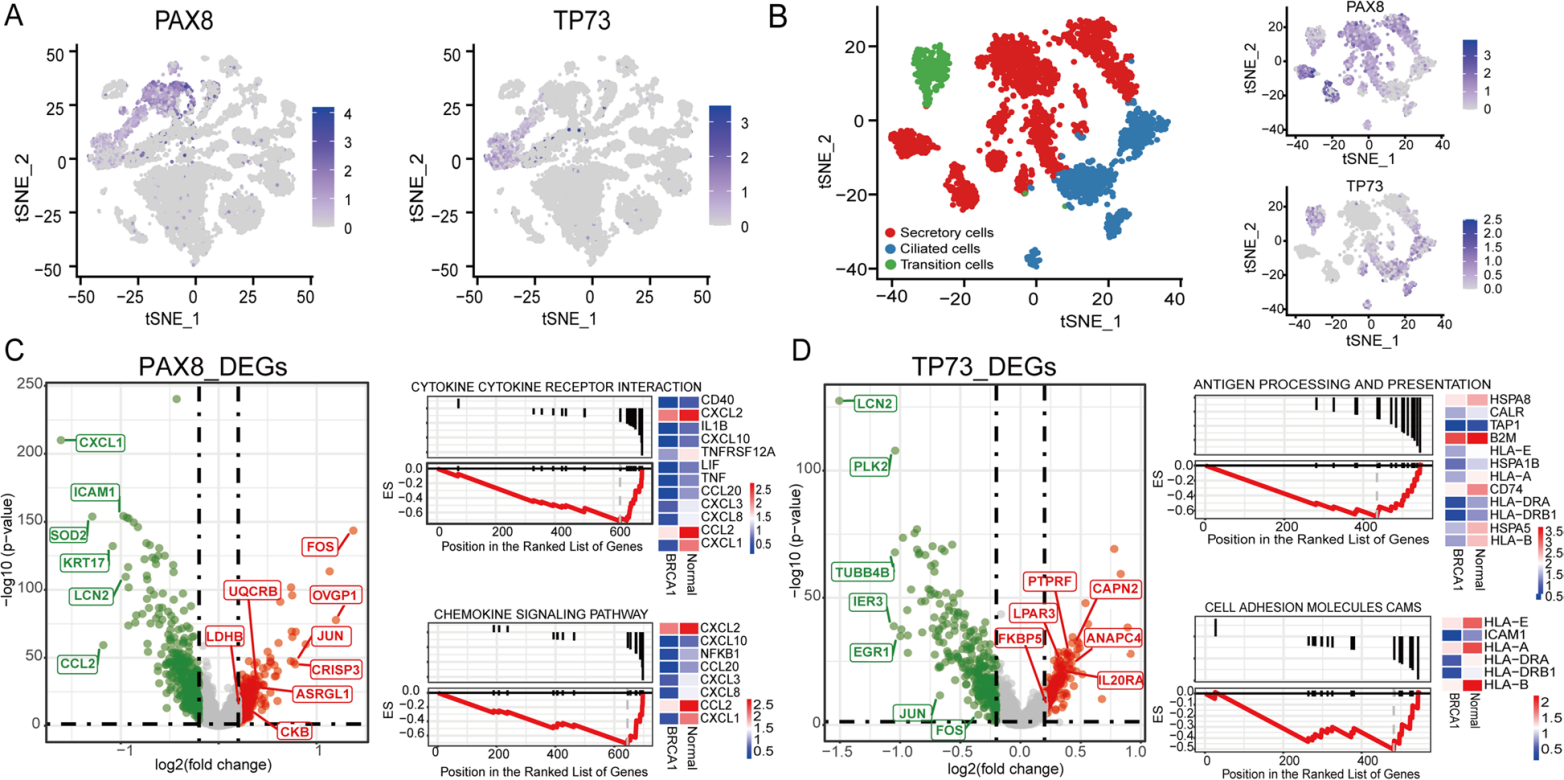
Tubal cellular populations and their differentially expressed genes. **A** Expression levels of a secretory marker, PAX8 (left panel), and a ciliated marker, TP73 (right panel). **B** tSNE plot shows the re-clustering result of all secretory and ciliated cells and the expression levels of their marker genes. **C** Volcano plot illustrating differential gene expressions between BRCA1 mutation carriers and normal controls in secretory cells (left panel), GSEA pathways enriched by differentially expressed genes (DEGs), the down-regulated pathways of cytokine-cytokine receptor interaction, and chemokine signaling pathway (right panel). **D** Volcano plot illustrating differential gene expression between BRCA1 mutation carriers and normal controls in ciliated cells (left panel), GSEA pathways enriched by differentially expressed genes, the down-regulated pathways of antigen processing and presentation and cell adhesion molecules cams (right panel). P values were estimated using the Benjamini-Hochberg procedure

Epithelial cells of the fallopian tube consist of ciliated and secretory cells; the latter are considered to be a potential cellular source of HGSC [12, 31]. It is known that the secretory cells highly express PAX8 [32], while TP73 is a specific marker of the ciliated cells [15]. The epithelial cells obtained from this study consisted of a continuum of cells expressing varying levels of these biomarkers, with two cell groups highly expressing either PAX8 or TP73, representing secretory and ciliated cells, respectively (Fig. 2A). We isolated these cells from the epithelial cell populations, and performed unsupervised reclustering. We also observed a cell population that expressed both PAX8 and TP73, known as “transition cells”. In total, there were 1990 secretory, 766 ciliated, and 277 transition cells (Fig. 2B). Moreover, the same t-SNE plot of these cells colored by sample source was reported in Fig. S2.

Compared to the secretory cells of the normal controls, *BRCA1* carriers differentially expressed a set of 697 genes (False Discovery Rate < 0.05, log2-fold change > 0.2 ). The top upregulated genes include oviductal glycoprotein 1 (OVGP1), cysteine-rich secretory protein 3 (CRISP3), and a critical component of the AP-1 transcription factor, FOS (Fig. 2C). OVGP1 is also called mucin 9 (MUC9), which can be secreted at increased levels in the serum of women with ovarian serous borderline tumors and low-grade serous cancers [33]. To explore functional changes of the secretory cells in *BRCA1* carriers, we performed GSEA (gene set enrichment analysis). Compared to normal controls, these cells significantly downregulated genes involved in cytokine-cytokine receptor interaction and chemokine signaling pathways (Fig. 2C). These changes could explain why we did not observe a high percentage of macrophages or T cells in the mutated tubal tissues, as the mutated epithelial cells may have suppressed inflammatory responses through gene regulation.

There were 550 differentially expressed genes identified in ciliated cells between *BRCA1* and non*-BRCA1*carriers. The ciliated cells of *BRCA1* carriers had higher levels of *CAPN2*, *PTPRF*, and *ANAPC4* gene expression, which are involved in cellular adhesion and the cell cycle. These ciliated cells were also enriched in genes involved in antigen processing and presentation (Fig. 2D). In contrast, secretory cells of the *BRCA1* carriers specifically upregulated some metabolism-associated genes, including *ASRGL1*, *MTF*, *CKB*, *UQCRB*, and *LDHB*. Importantly, *UQCRB* is related to oxidative phosphorylation and *LDHB* is involved in glycolysis. Furthermore, in *BRCA1* carriers, both *FOS* and *JUN* were downregulated in the ciliated cells and upregulated in the secretory cells. The joint upregulation of both AP-1 components (*FOS* and *JUN*) in the secretory cells could explain why these cells have a higher potential for the malignant transformation and migration in BRCA1 carriers compared to that in non-*BRCA1* carriers [34].

### Subset of secretory cells of BRCA1 carriers exhibited EMT phenotype

**Fig. 3:**
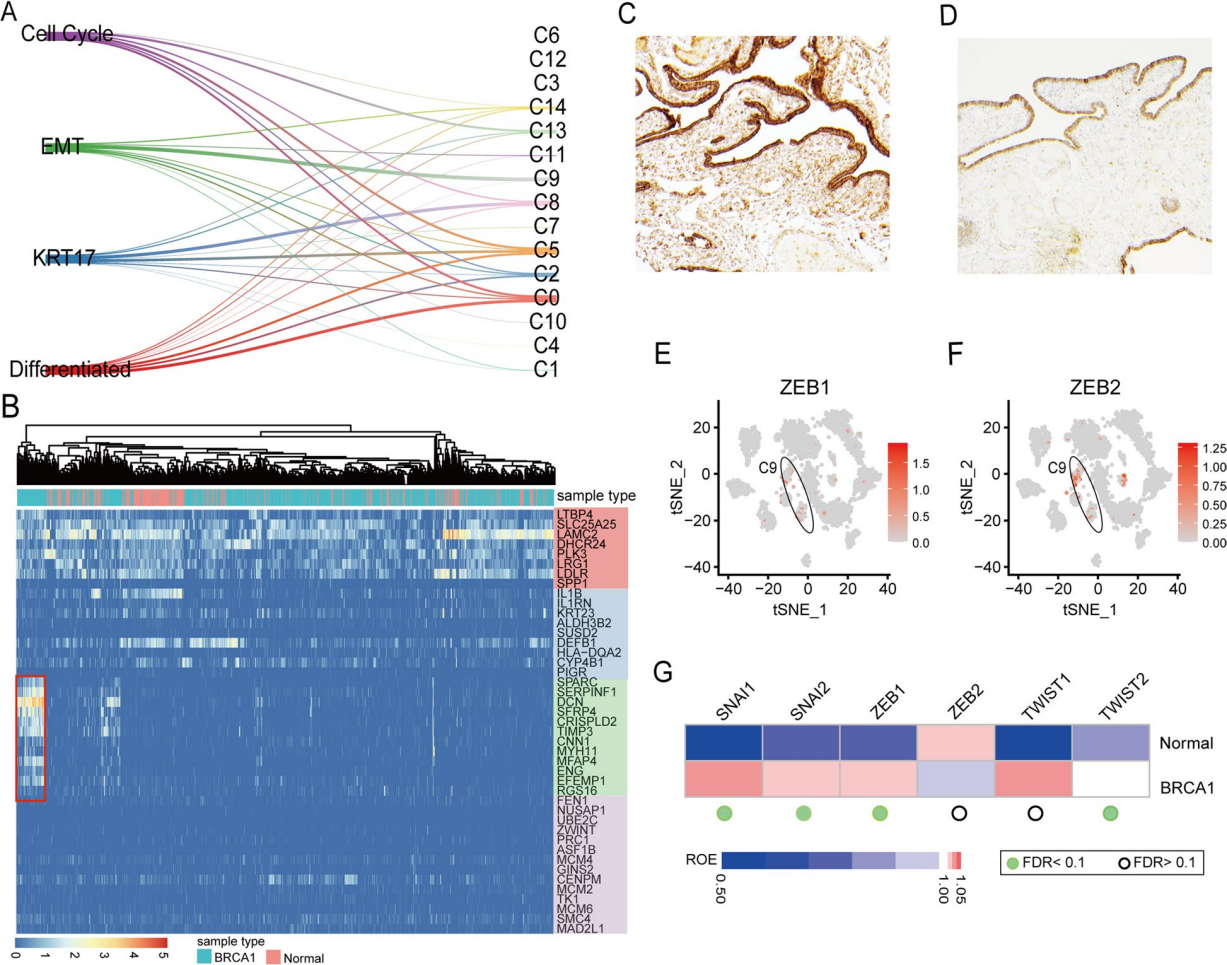
EMT phenotype in tubal secretory cells and fibroblast. **A** The similarities between 4 major secretory cell subtypes and our epithelial cell clusters, the width of line describes the value of similarity scores. **B** Heatmap shows the expression level of secretory cell subtype signatures in all secretory cells from six samples, and the EMT signature highly expressed in 126 single cells (indicated by red box) from one BRCA1 mutation carrier. **C** IHC staining confirms the existence of EMT cluster by its markers SFRP4 in epithelial and stromal compartments of a tubal section from a BRCA1 carrier (BRCA1_1) and **D** non-BRCA1 carrier (Normal_3). **E**, **F** Expression levels of EMT-related transcriptional factors ZEB1 and ZEB2 in epithelial cell clusters. **G** The distribution of EMT-TF in fibroblast of BRCA1 mutation carriers and normal controls, heatmap shows the ratio of observed cell number over the expected cell number of EMT-TF expressed cells, the dots describe the adjusted p value (FDR) of chi-squared test, which were estimated using the Benjamini-Hochberg procedure

A recent study identified 4 major clusters of secretory cell subtypes (cell-cycle, EMT, differentiated, and KRT17 clusters) in FTE with HGSC based on single-cell sequencing experiments [24]. The EMT cluster was associated with a high expression of RGS16 (Regulator of G Protein Signaling), and genes enriched in the extracellular matrix pathway. They linked these subtypes of the secretory cells to HGSC status by subtype signatures and found that the EMT-high HGSC subtype was strongly associated with the worst prognosis [24]. We sought to explore if similar cell subtypes were present in “normal” looking tubal epithelia from *BRCA1* mutation patients. To do so, we normalized our data and evaluated the similarities between the epithelial cell clusters and these four secretory cell subtypes (Fig. 3A) by using mutual nearest neighbor method [35]. Of the 15 epithelial clusters identified in this study (C0–C14), we noticed that C9 showed the highest similarity with the EMT-subtype. The C9 cluster, containing 133 cells, was identified in one *BRCA1* carrier (BRCA1_1). In addition, when comparing the secretory cell subtype gene signatures to the data we obtained, we confirmed 113 out of the 133 cells highly expressed EMT markers, while no such cells were found in the normal controls (Fig. 3B). The differences were statistically significant (Fisher test *p* < 2.2 × 10^−16^). We performed IHC staining of two putative EMT biomarkers, SFRP4 and DCN using the Formalin-Fixed Paraffin-Embedded (FFPE) samples from the *BRCA1* mutation patients in our cohort. We observed high expression of both markers in the epithelial cells as well as the stroma of the fallopian tube samples (Fig. 3C, D; Table S4), we also showed IHC staining results of other samples in Fig. S3. Specifically, the protein expression levels of SFRP4 and DCN were confirmed in all 3 BRCA1 mutation patients, suggesting that the EMT phenotype observed for BRCA1_1 in the single cell data was not an isolated case (Table S4). However, the staining of SFRP4 and DCN on the corresponding tubal sections was higher in BRCA1_1 than that of the other two cases, which explained why we did not observe a strong EMT signature in BRCA1_2 and BRCA1_3 at the mRNA level in our computational analysis.

We specifically checked the expression level of putative EMT-related transcriptional factors (not included in the above signatures) in all epithelial cells, including SNAI1, SNAI2, ZEB1, ZEB2, TWIST1, and TWIST2. Consistent with the above results, only the C9 cluster highly expressed ZEB1 and ZEB2 (Fig. 3E–F), while no elevated expression of EMT-related transcriptional factors was observed in other clusters. These findings indicate that a subset of secretory cells from *BRCA1* carriers likely undergo an EMT process prior to its transformation into the malignant phenotype and that this transition may be initiated by activating *FOS/JUN* genes.

In addition to the secretory cells, we also observed EMT-related characteristics in the fibroblasts of the *BRCA1* carriers, which were not seen in the control tubal tissue. It is worthwhile to mention that EMT-related gene expression features are commonly present in cancer-associated fibroblasts [36]. Compared to the normal controls, the fibroblasts of *BRCA1* mutation carriers were significantly enriched with cells expressing EMT-TF, including *SNAI1*, *SNAI2*, *ZEB1*, and *TWIST2* (chi-squared test FDR < 0.1, Fig. 3G and Table S5). Fibroblast-specific re-clustering analysis showed that the subsets highly expressed *CXCL12* and *IL6* genes (Fig. S4), which are known to regulate inflammatory responses and activate *JAK/STAT* signaling pathways in HGSC [37, 38]. Together, our findings reveal a novel and sophisticated interplay between the tubal secretory cells and the fibroblasts of sub-epithelial stroma in *BRCA1* mutation carriers at a pre-malignant phase. This may explain the tendency of tubal epithelial cell detachment in *BRCA1* carriers compared to normal controls, as well as the early spreading behavior of HGSC when a malignant transformation is in play.

### Phenotypic diversity of EMT-featured secretory cells in BRCA1 carriers

**Fig. 4:**
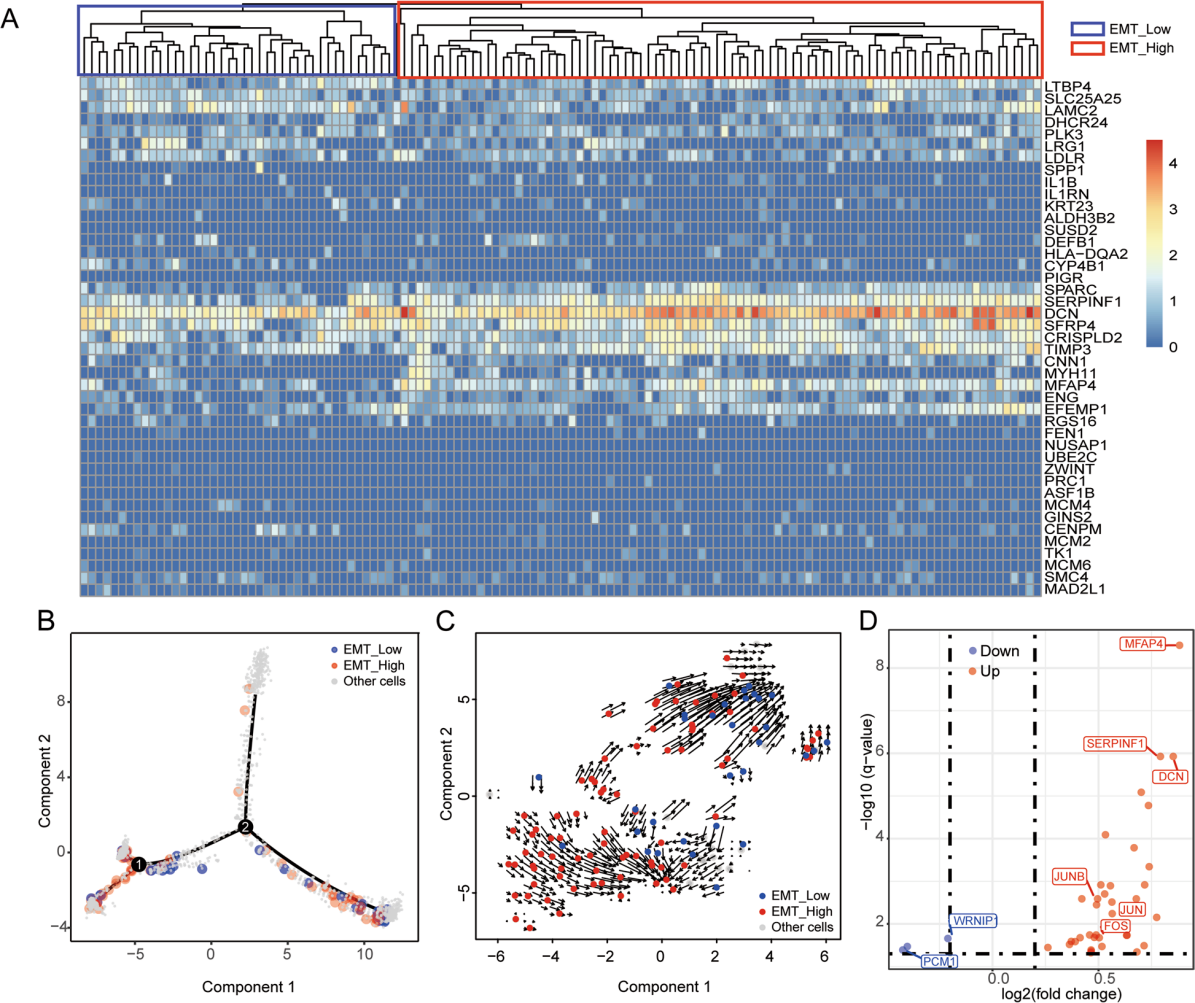
Sub-populations of EMT-featured secretory cells. **A** Heatmap and hierarchical clustering of 126 cells expressed EMT signature. **B** The branched trajectory of secretory cell status transition in a two-dimensional state-space inferred by Monocle 2 in all samples. Each dot corresponds to one single cell, colored according to cell types. **C** Visualization of the RNA velocity analysis results on the tSNE plot of the tubal secretory cells. **D** Volcano plot illustrating differential gene expression between EMT-high and EMT-low cells with red dots representing upregulated while blue dots for downregulated genes. FDR q values were estimated using the Benjamini-Hochberg procedure

Having established the presence of EMT secretory cells in *BRCA1* carriers, we observed a previously unreported phenotypic split in this group of 126 cells. After unsupervised clustering, we discovered two distinct groups based on gene expression patterns and named them EMT-low (41 cells) and EMT-high (85 cells) secretory cells (Fig. 4A). Compared to the EMT-low group, the expression level of EMT signature genes including *SERPINF1* and *DCN* was significantly higher in the EMT-high group. To investigate their evolutionary relationships, we constructed a single-cell trajectory using the data from all the secretory cells from *BRCA1* carriers and non-*BRCA1* carriers [39]. Surprisingly, although both groups were phenotypically assigned to C9, EMT-high cells were significantly enriched in the left branch, while EMT-low cells clustered in the right (Fig. 4B). This separation might suggest different evolutionary termini of the two groups.

To further evaluate these findings, we examined the cell expression dynamics by using RNA velocity [40], which predicts the cell’s future transcriptional state by computing the ratio of unspliced to spliced mRNA transcripts. We visualized the transcriptional profile of the C9 cells by mapping them onto a vector field, with vectors representing the polarity and speed of cell differentiation (Fig. 4C). Similar to the trajectory analysis, we observed two separate EMT cell populations, with speed vectors pointing primarily upwards in the EMT-low cell population and downwards in the EMT-high cell population. This polarity suggests that the two groups of cells were actively progressing towards distinct future states. When comparing the expression profiles of EMT-high and EMT-low, *JUN* and *FOS* again appeared as the top upregulated genes in the EMT-high group (Fig. 4D). It has been reported that the cooperation of the EMT inducer *ZEB1* and AP-1 factor *JUN* may drive oncogenesis [41]. Based on these findings, we speculate that the EMT-high secretory cells may represent the direct progenitor cells of HGSC, while EMT-low cells might be outcompeted during tumor progression associated evolutionary processes, and thus not observed in the developed HGSCs, as reported recently [24].

### Clonal expansion and functional exhaustion of T cells in BRCA1 carriers

**Fig. 5:**
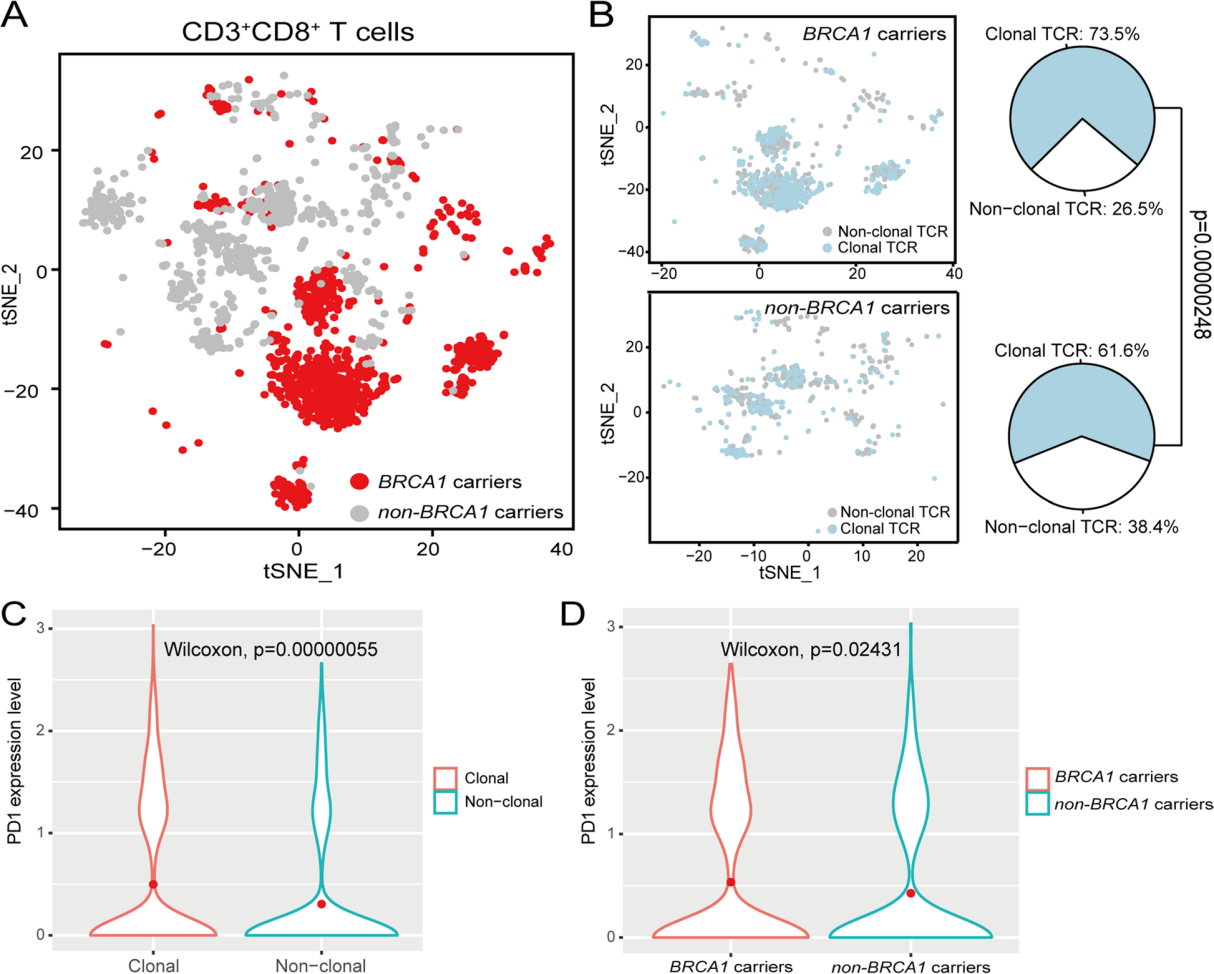
Profiling T cells in BRCA1 carriers. **A** t-SNE plot displaying the CD3^+^CD8^+^ T cells in BRCA1 and non-BRCA1 carriers. **B** t-SNE plot showing the distribution of clonal TCRs and pie chart showing the percentage of clonal TCRs in BRCA1 and non-BRCA1 carriers. **C** Violin plot showing the PD1 expression level in clonal and non-clonal CD8+ T cells. **D** Violin plot showing the PD1 expression level in BRCA1 mutation carriers and normal controls for clonal CD8+ T cells. Statistical significance was estimated using a two-sided Wilcoxon rank-sum test

Our single cell analysis captured 3243 T cells in the fallopian tube samples, comprising 17.1% of the total cell population. In order to investigate the dynamics of T cells in the microenvironment of *BRCA1* carriers and normal controls, we performed targeted V(D)J capture to obtain the single cell TCR sequences (scTCR-seq) from all six samples. 2,410 T cells were matched with paired TCR sequences and were used for downstream analysis. There were totally 1,647 CD8^+^ T cells in six samples. Importantly, we observed significantly enriched CD8^+^ T cells in *BRCA1* carriers (*χ*^2^ test *p* = 4.07 × 10^−8^), suggesting an early role for the adaptive immune system prior to tumor initiation (Fig. 5A). We also showed the t-SNE plot of CD8^+^ T cells colored by distinct samples in Fig. S5. We next used paired TCR sequences as clonal markers to trace the expansion of T cells. ‘Clonal’ was defined as more than one T cell carrying identical pairs of TCRs. Among 1,461 CD8^+^ T cells with full length productive CDR3 sequences for both α and β chains, we identified 1,009 clonal CD8^+^ T cells and 452 non-clonal CD8^+^ T cells. Of these, 672 (73.5%) clonal CD8^+^ T cells and 242 (26.5%) non-clonal CD8^+^ T cells were found in the *BRCA1* mutation carriers, with the percentage of clonal CD8^+^ T cells significantly higher than normal controls (*χ*^2^ test *p* = 2.48 × 10^−6^, Fig. 5B).

We then determined the extent of T cell exhaustion by using putative T cell exhaustion markers [42] and calculated the number of cells expressing PD-1 (PDCD1), TIM3 (HAVCR2), LAG3, CTLA4, TOX, and ENTPD1 (CD39) in *BRCA1* carriers and non-*BRCA1* carriers, respectively. We used the ratio of observed over expected cell numbers (ROE) to measure the enrichment of exhaustion markers in the study and control samples [43]. PD-1, LAG3, TOX, and CD39 exhausted T cells were significantly enriched in *BRCA1* carriers (*χ*^2^ test *p* = 0.026, *p* = 0.0015, *p* = 4.715 × 10^−6^ and *p* = 1.97 × 10^−13^, respectively). We further examined the expression of these T cell exhaustion markers and only observed significantly higher PD-l expression in clonal CD8^+^ T cells compared with non-clonal CD8^+^ T cells (Wilcoxon test *p* = 5.5 × 10^−7^) (Fig. 5C). Among these clonal CD8^+^ T cells, the PD-1 positivity was significantly increased in *BRCA1* carriers compared with the normal controls (Wilcoxon test *p* = 0.02431) (Fig. 5D), while other T cell exhaustion markers did not exhibit the similar tendency. These observations strongly suggest an early T cell exhaustion phenotype in the microenvironment of *BRCA1* mutated fallopian tubal mucosae, which may provide an explanation for the expedited development of HGSC among patients with BRCA1 germline mutations.

## Discussion

In this work, we performed single-cell RNA and TCR sequencing on FTE cells from 3 *BRCA1* carriers and 3 wild-type controls. The results showed significant differences between the tubal secretory cell populations and microenvironments in the *BRCA1* and non-*BRCA1* carriers, providing explanation, at least in part, as to why patients with *BRCA1* germline mutations have such a significantly increased risk of HGSC development. To our knowledge, this study is the first addressing such differences by using normal appearing tubal mucosae from patients with and without *BRCA1* germline mutations.

Although there is evidence that tubal secretory cells contribute to the development of ovarian HGSC, it is unclear what molecular changes among secretory and other cell types drive this process in *BRCA1* carriers. Furthermore, adaptive immunity in non-cancerous tubal epithelial cells of HGSC patients remains poorly understood. With single-cell RNA-seq and TCR-seq, we characterized the T cells from patients with and without *BRCA1* mutations. The fact that CD8^+^ T cells were both enriched and clonally expanded in the *BRCA1* mutation carriers suggests that there is an early adaptive immune response among the FTE cells in patients with a BRCA1 mutation, even before the development of cancer. Based on these findings, we hypothesize that such early adaptive immune response is induced by the EMT process. This hypothesis is supported by the presence of EMT-featured tubal secretory cells and fibroblasts as discussed above. It is further supported by the high expression of the T cell activation and exhaustion marker PD-1 in the clonally expanded CD8^+^ T cells. These findings suggest that immune evasion within tubal mucosae may represent an early event contributing to the initial process of HGSC development.

A recent study addressing non-genetic heterogeneity of ovarian serous cancers [24] used single-cell RNA sequencing to show that FTE contain 4 secretory cell subtypes with distinct molecular signatures. After deconvoluting the expression data obtained in that study, they were able to identify distinct FTE subtypes within ovarian serous cancers that correlated with patient survival [24]. Notably, an EMT-high subtype of tubal secretory cells that was enriched in genes related to the mesenchymal subtype of HGSC was associated with poor clinical prognosis compared to non-EMT-high subtypes [24]. This observation is in line with the previous finding that prominent EMT processes in human cancers enhance cancer invasion and drug resistance [44]. In the current study, we observed these EMT features in tubal secretory cells from a benign fallopian tube from one of the 3 BRCA1 carriers (BRCA1_1), who had a specific BRCA1 c.68_69dup (p.Cys24fs) mutation. However, this frameshift insertion was not identified in other two *BRCA1* carriers. It is currently unclear if this specific frameshift mutation is more prone to the HGSC development than those *BRCA1* carriers without such mutation. Future studies to better define the relationship between specific BRCA1 mutations and biologic and clinical outcomes will help improve our understanding of who is at the highest risk of developing HGSC and why.

The EMT-featured secretory cells from BRCA1_1 had upregulated expression of *FOS* and *JUN* of AP-1 complex and the two EMT-TFs ZEB1 and ZEB2. However, we couldn’t rule out the possibility that secretory cells were more prone to stress response during cell preparation might still exist. A previous study, consistent with the findings of the current study, showed that EMT can be induced by multiple extracellular signals related to the activation of a plethora of EMT-TFs that belong to different genes including SNAIL, TWIST, and ZEB families [45]. In addition, intra-cancer heterogeneity is a well-recognized phenomenon and different levels of EMT-TFs may be present in tumor tissue sections from a single cancer case [45]. It is known that aberrant activation of EMT-TFs in human neoplastic epithelial cells can promote cancer cell plasticity and trigger the tumor initiation process [46]. Our results indicated that EMT happens even in normal tubal epithelium, prior to the formation of HGSC or its precursor STIC; this transition could be associated with changes in the immune environment that explain the high metastatic potential of the even early-stage ovarian HGSC. Interestingly, there were two distinct cell groups, EMT-high and EMT-low, from EMT-featured secretory cells of the BRCA1_1 patient showing bifurcated evolutionary termini and future cellular lineage states. Importantly, JUN and FOS again appeared as the top upregulated genes in the EMT-high group compared to that of EMT-low group. Such findings suggest that EMT-high secretory cells may represent the progenitor cells of HGSC, while the EMT-low secretory cells may be outcompeted and subsequently not participate in the process of tumor initiation and development.

Cancer tissues are composed of cancer cells and adjacent fibroblasts containing connective tissue. As mentioned above, EMT plays a role in cancer development. Recently, research have shown that cancer-associated fibroblasts (CAF) are a major component of the cancer mesenchyme, and they are closely related to the EMT process in various cancers [37]. Interactions between CAFs and cancer epithelial cells play a role in promoting cancer development and progression [36]. Results of the current study showed that the fibroblasts of *BRCA1* carriers expressed a high level of EMT-TFs, including SNAI1, SNAI2, ZEB1, and TWIST2. There was a subset of fibroblasts showing a high level of essential chemokine and cytokine expression including *CXCL12* and *IL6*. Previous studies have demonstrated that CAF-derived CXCL12 induces EMT through the CXCR4/Wnt/β-catenin pathway in ovarian epithelial cancers [47] and that IL-6 secreted by CAFs can enhance EMT in lung cancer cells [48] and activate *JAK/STAT* signaling pathways in HGSC [37, 38]. Put together, we believe there may be an interaction between tubal secretory cells and fibroblasts promoting the EMT process of secretory cells within the tubal mucosae of patients with *BRCA1* germline mutations, and such interaction may facilitate the initiation of the HGSC development.

This study has several clinical implications. There are two identified biomarkers, CRISP3 and MUC9, that might be used for ovarian cancer early diagnosis and prevention. Both genes showed a higher level of expression in *BRCA1* carriers, and their translational products are secreted proteins. Of them, MUC9 has been reported in a previous study to be upregulated in early-stage ovarian cancers. The study also revealed a number of clonally expanded T cell receptors that might be used to identify new HGSC patients by using the TCR repertoire data [49]. These novel findings, if further confirmed by additional studies, may aid in generating effective early detection and prevention strategies for ovarian HGSC. Aberrant expression of immune-related cellular markers disclosed in this study may be used in clinic to aid in screening and identifying patients who will benefit the most from a risk-reducing salpingo-oophorectomy. In addition to the above-mentioned biomarkers, EMT-related molecular changes may serve the same purpose. Studies identifying *BRCA1* carriers with a true high-risk for HGSC development are currently ongoing in our laboratories within UTSW Medical Center.

Our study, however, does have a few limitations. First, due to the low incidence of *BRCA1* germline mutations, the sample size of the *BRCA1* carriers is small, which might limit the discovery power. Therefore, future studies with samples from more patients with germline *BRCA1* mutations may help to determine the cause-and-effect relationships between germline *BRCA1* mutations and ovarian cancer development. Second, mechanistic investigation of the EMT process of secretory cells with *BRCA1* germline mutations could reveal more molecular targets against HGSCs. This would likely require a suitable animal model or cell lines that do not yet exist. In addition, the other BRCA1 mutations or other coding gene mutations would be also useful for characterizing the EMT process. Third, in this study, we only observed one patient with strong EMT signatures in the epithelial cells; however, this could be related to the incomplete penetrance of BRCA gene mutations [7–10]. Finally, our observation of PD-1 expression on the T cells requires further verification using more patient samples. More mechanistic investigation on the infiltrating T cells in the pre-cancerous lesions is also required to establish the early exhaustion phenotype, as PD-1 expression is an indicator of T cell activation.

To overcome these limitations, we could collect more patients with germline BRCA1 mutations in the future to perform multi-omics single-cell sequencing and determine early-stage events of ovarian cancer in BRCA1 carriers. Furthermore, we could use the early-stage HGSC cell line to demonstrate the EMT transition in secretory cells, block the EMT process by silencing EMT-TFs and characterize transcriptome of these cells until the early-stage HGSC cell lines are available. These future directions would help us to deeply understand the mechanism of early-stage changes of ovarian cancer.

In summary, despite imperfections, findings of the current study illuminate the cellular landscape of fallopian tube mucosae in *BRCA1* carriers. We identified EMT-high and EMT-low secretory cell subgroups. We believe the former have the growth advantage leading to the development of HGSC, while the latter is likely to disappear in the process of HGSC development. In addition, the clonally expanded CD8^+^ T cells associated with elevated PD-1 expression indicate that immune evasion may occur within normal-looking tubal mucosae in *BRCA1 mutation* carriers. Therefore, EMT featured tubal secretory cells and immune evasion in tubal mucosae may represent two previously unrecognized early events initiating the tumor growth and development processes. These novel findings may aid in generating effective early detection and prevention strategies for ovarian HGSC.

## Conclusions

In summary, our research provides a probable molecular changes explaining why some women with *BRCA1* germline mutation present with early development and rapid dissemination of the HGSC; these molecular changes may be beneficial in identifying particularly high-risk patients with *BRCA1* germline mutations.

## Supplementary Information

**Additional file 1: Figure S1.** Two-dimensional t-SNE projection of 19,008 cells from fallopian tube of 6 samples. Distinct colors indicate the cells from different samples. **Figure S2.** Two-dimensional t-SNE projection of 3033 secretory and ciliated cells from 6 samples. Distinct colors indicate the cells from different samples. **Figure S3.** IHC staining confirms the existence of EMT cluster by its markers SFRP4 in epithelial and stromal compartments of a tubal section from BRCA1_2 (A), BRCA1_3 (B), Normal_1 (C) and Normal_2 (D).

**Additional file 2: Table S1.** Patient information.

**Additional file 3: Table S2.** Cell numbers and percentages of each major cell type.

**Additional file 4: Table S2.** Top 100 marker genes of fibroblast, T cell, monocytes and blood vessel endothelium.

**Additional file 5: Table S4.** IHC staining results of SFRP4 and DCN.

**Additional file 6: Table S5.** The ROE of all TFs in BRCA1 and Normal sample.

## Data Availability

All data generated in this study are available at Mendeley Data, with DOI: 10.17632/66fz3h924x.1, the link of the data is https://data.mendeley.com/datasets/66fz3h924x/1.

## Abbreviations

HGSC: High-grade serous carcinoma
EMT: Epithelial-to-mesenchymal transition
OEC: Ovarian epithelial cancer
STIC: Serous tubal intraepithelial carcinoma
STIL: Serous tubal intraepithelial lesions
SCOUT: Secretory cell outgrowth
SCE: Secretory cell expansion
FTE: Fallopian tube epithelia
scRNA-seq: Single-cell RNA sequencing
CAF: Cancer-associated fibroblasts
EMT-TFs: EMT-transcription factors
CCA: Canonical correlation analysis
FC: Fold change
t-SNE: T-distributed stochastic neighbor embedding
GSEA: Gene set enrichment analysis
FFPE: Formalin-Fixed Paraffin-Embedded
scTCR-seq: Single-cell TCR sequences
ROE: Ratio of observed over expected cell numbers
GSEA: Gene set enrichment analysis
