# Supplementary material for: Dissection of transcriptome dysregulation and immune characterization in women with germline BRCA1 mutation at single-cell resolution
Source: BMC Med. 2022 Sep 9;20:283. doi: 10.1186/s12916-022-02489-9 (PMC9461201; doi:10.1186/s12916-022-02489-9)
Supplement: Supplementary file 1 — Additional file 1: Figure S1. Two-dimensional t-SNE projection of 19,008 cells from fallopian tube of 6 samples. Distinct colors indicate the cells from different samples. Figure S2. Two-dimensional t-SNE projection of 3033 secretory and ciliated cells from 6 samples. Distinct colors indicate the cells from different samples. Figure S3. IHC staining confirms the existence of EMT cluster by its markers SFRP4 in epithelial and stromal compartments of a tubal section from BRCA1_2 (A), BRCA1_3 (B), Normal_1 (C) and Normal_2 (D). [file 12916_2022_2489_MOESM1_ESM.docx]

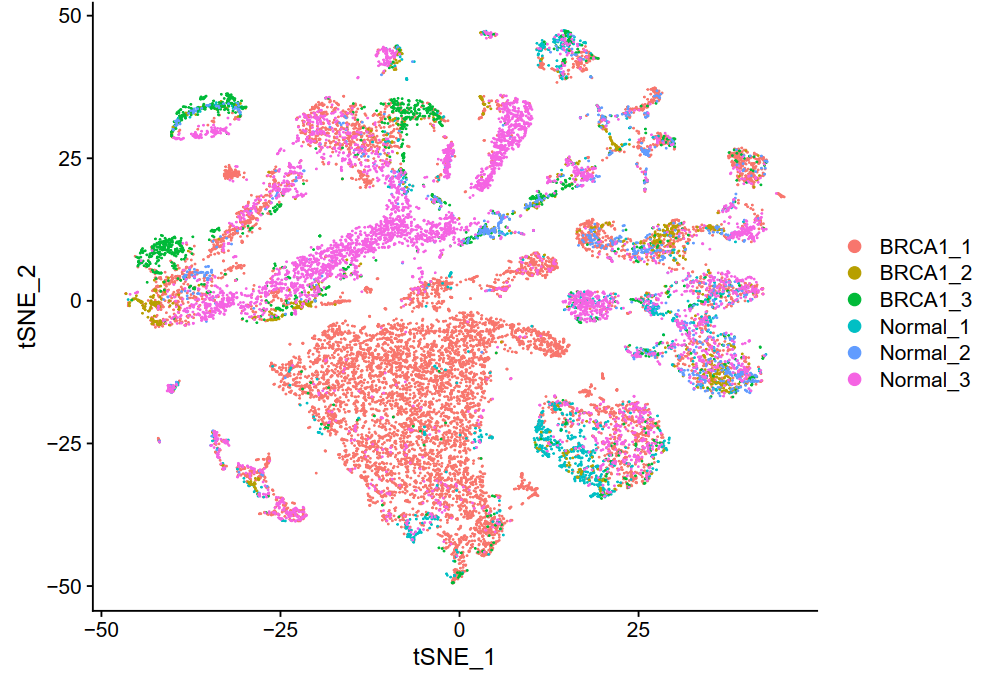


Fig. S1. Two-dimensional t-SNE projection of 19,008 cells from fallopian tube of 6 samples. Distinct colors indicate the cells from different samples.


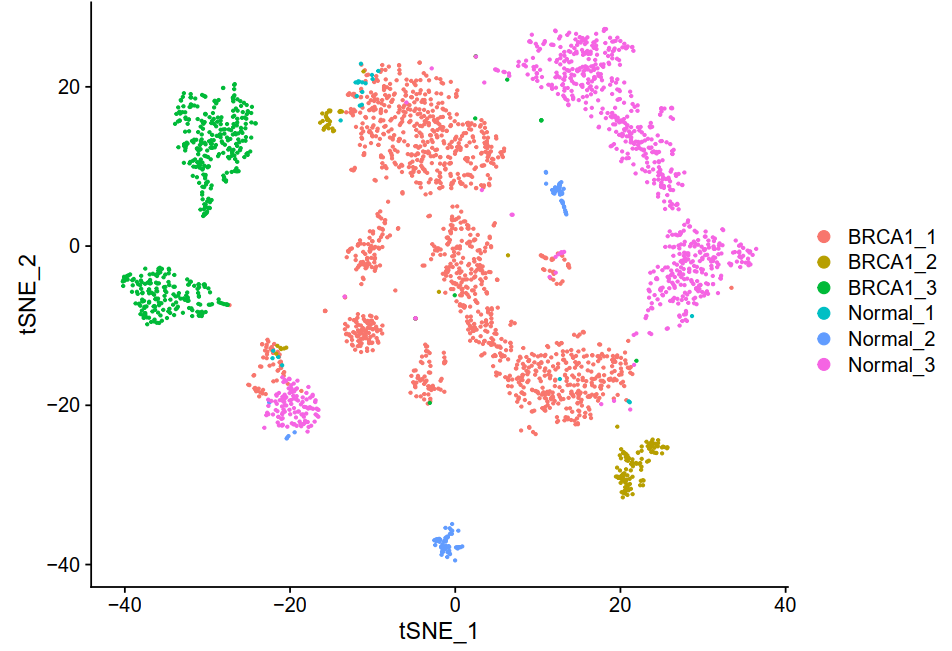


Fig. S2. Two-dimensional t-SNE projection of 3033 secretory and ciliated cells from 6 samples. Distinct colors indicate the cells from different samples.


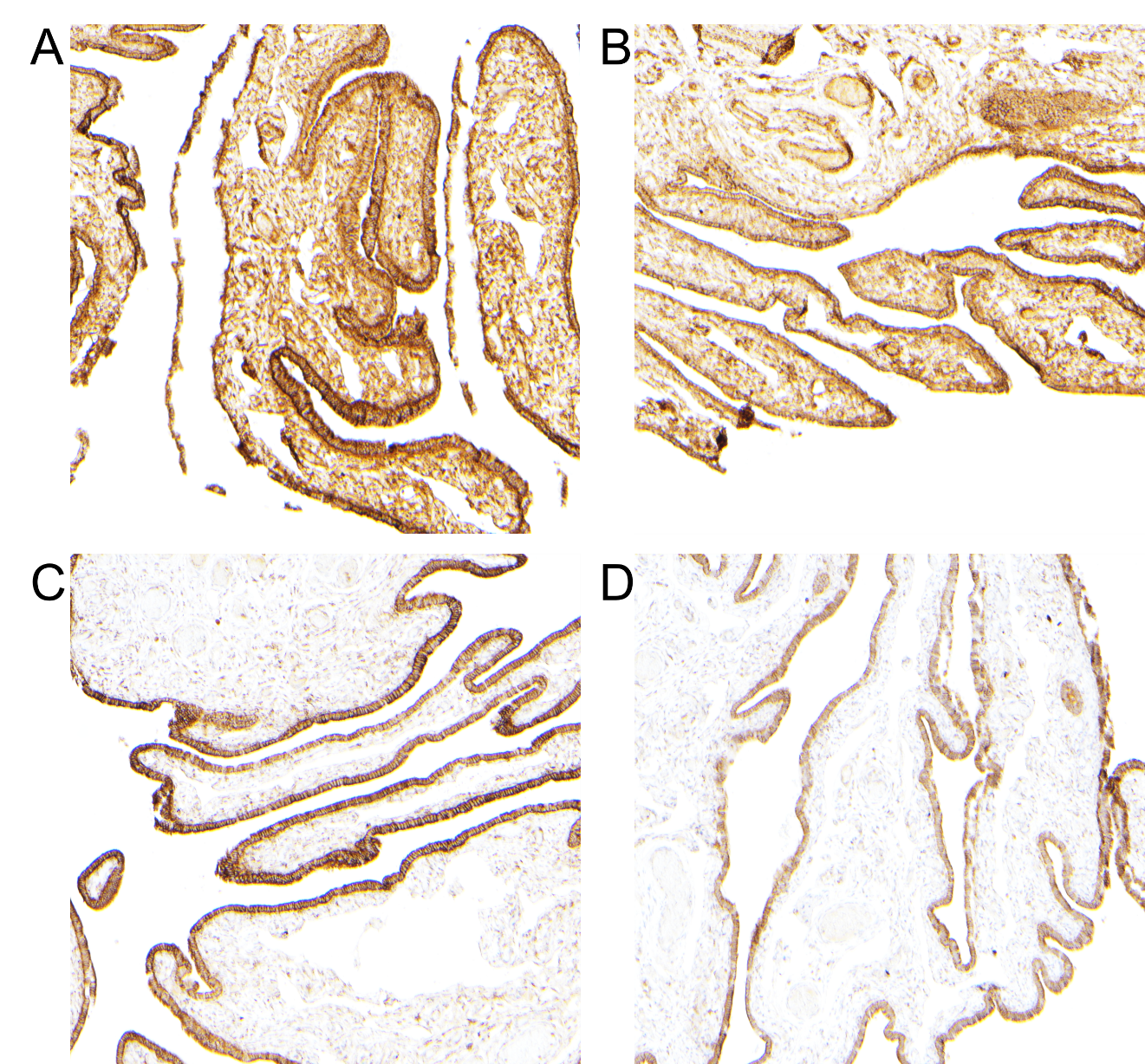


Fig. S3. IHC staining confirms the existence of EMT cluster by its markers SFRP4 in epithelial and stromal compartments of a tubal section from BRCA1_2 (A), BRCA1_3 (B), Normal_1 (C) and Normal_2 (D).


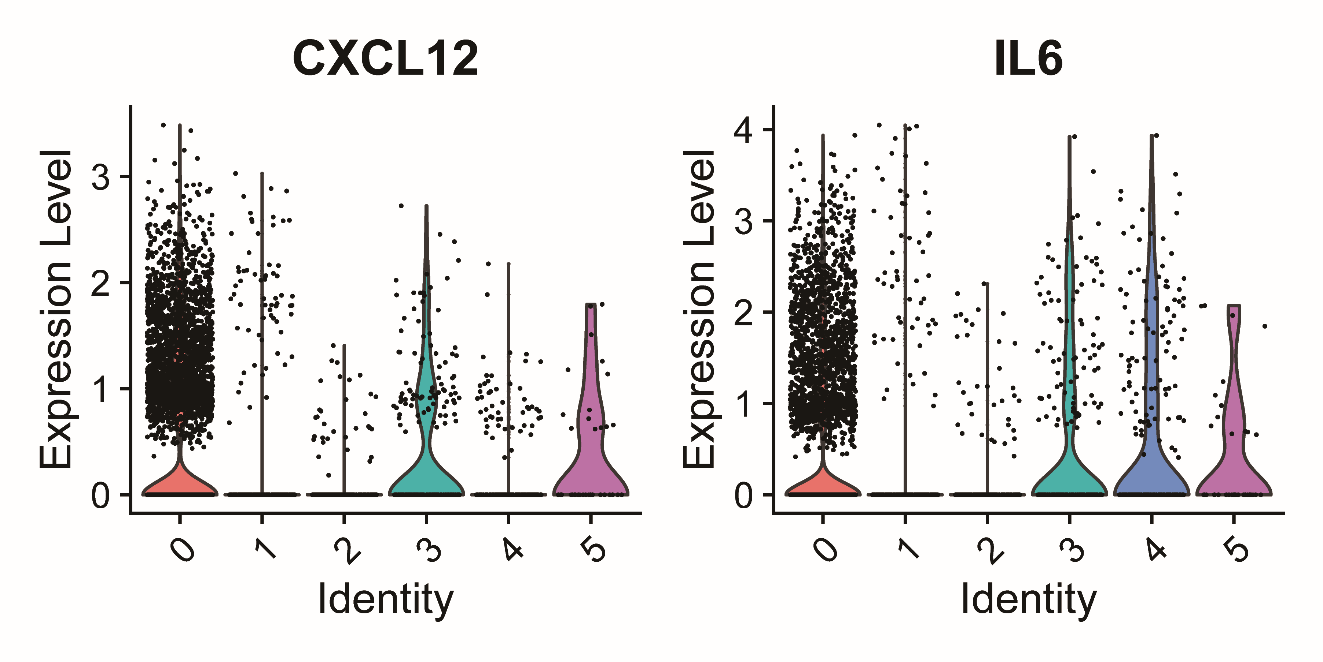


Fig. S4. mRNA expression levels of CXCL12 and IL6 are shown in violin plots for all six fibroblast clusters (fibroblast cluster 0 to 5, from left to right).


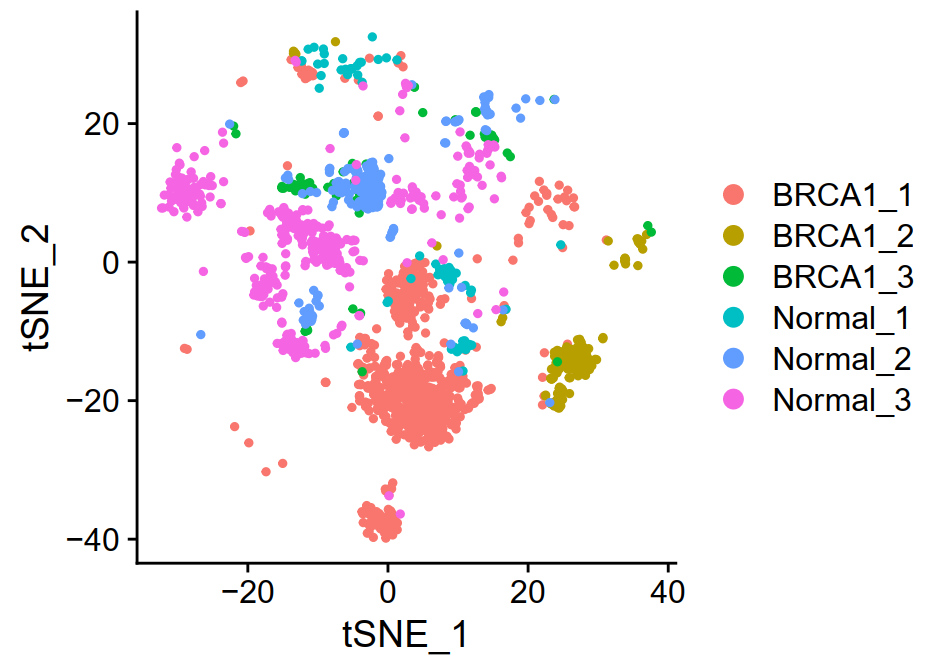


Fig. S5. Two-dimensional t-SNE projection of 1,647 CD8+ T cells from 6 samples. Distinct colors indicate the cells from different samples.
